# Powerful Sequence Similarity Search Methods and In-Depth Manual Analyses Can Identify Remote Homologs in Many Apparently “Orphan” Viral Proteins

**DOI:** 10.1128/JVI.02595-13

**Published:** 2014-01

**Authors:** Durga B. Kuchibhatla, Westley A. Sherman, Betty Y. W. Chung, Shelley Cook, Georg Schneider, Birgit Eisenhaber, David G. Karlin

**Affiliations:** a Bioinformatics Institute (BII), A*STAR (Agency for Science, Technology and Research), Matrix, Singapore; b Department of Plant Sciences, University of Cambridge, Cambridge, United Kingdom; c Life Sciences—Parasites and Vectors Division, Natural History Museum, Cromwell Road, London, United Kingdom; d IST Austria (Institute of Science and Technology Austria), Klosterneuburg, Austria; e Department of Zoology, Oxford University, Oxford OX1 3PS, United Kingdom; f Division of Structural Biology, Oxford University, Oxford OX1 7BN, United Kingdom

## Abstract

The genome sequences of new viruses often contain many “orphan” or “taxon-specific” proteins apparently lacking homologs. However, because viral proteins evolve very fast, commonly used sequence similarity detection methods such as BLAST may overlook homologs. We analyzed a data set of proteins from RNA viruses characterized as “genus specific” by BLAST. More powerful methods developed recently, such as HHblits or HHpred (available through web-based, user-friendly interfaces), could detect distant homologs of a quarter of these proteins, suggesting that these methods should be used to annotate viral genomes. In-depth manual analyses of a subset of the remaining sequences, guided by contextual information such as taxonomy, gene order, or domain cooccurrence, identified distant homologs of another third. Thus, a combination of powerful automated methods and manual analyses can uncover distant homologs of many proteins thought to be orphans. We expect these methodological results to be also applicable to cellular organisms, since they generally evolve much more slowly than RNA viruses. As an application, we reanalyzed the genome of a bee pathogen, Chronic bee paralysis virus (CBPV). We could identify homologs of most of its proteins thought to be orphans; in each case, identifying homologs provided functional clues. We discovered that CBPV encodes a domain homologous to the Alphavirus methyltransferase-guanylyltransferase; a putative membrane protein, SP24, with homologs in unrelated insect viruses and insect-transmitted plant viruses having different morphologies (cileviruses, higreviruses, blunerviruses, negeviruses); and a putative virion glycoprotein, ORF2, also found in negeviruses. SP24 and ORF2 are probably major structural components of the virions.

## INTRODUCTION

The detection of distant homologs of a protein has many applications. For example, it can provide clues to its function, guide the choice of substitutions for experimental studies, and facilitate three-dimensional (3D) structure determination (1). However, several sequence-based studies have reported that a significant fraction of viral proteins had no detectable homologs (2–4). These proteins have been called “orphans,” “ORFans” (5), or more accurately, “taxonomically restricted” (6) to indicate that they have no detectable homologs outside a certain taxon.

Some proteins classified as taxonomically restricted are thought to be truly specific to a particular organism, which they may endow with unique functions (6, 7). However, given the high rate of evolution of viral proteins, especially in RNA viruses (8), some orphans may in fact be part of larger protein families whose other members have diverged in sequence beyond recognition (9, 10). In fact, studies of viral orphans have relied mainly on the BLAST (Basic Local Alignment Search Tool) program (11, 12) to identify homologs (2, 3), rather than on more recent, powerful methods based on sequence profiles, such as sequence-profile comparison (PSI-BLAST [11], HMMER3 [13]) or profile-profile comparison (HHpred [14], HHblits [15], FFAS [1], WebPRC [16]). In contrast to BLAST, which compares single sequences, these methods rely on the comparison of multiple-sequence alignments, encoded as sequence profiles. A sequence profile is a representation of a multiple-sequence alignment that contains information about which amino acids are “allowed” at each position of the alignment and with what probability (17). Comparing profiles is much more sensitive than comparing single sequences, because the profiles contain information about how the sequences can evolve and can thus identify faint similarities that remain after the sequences have evolved apart (18, 19).

In the first part of this study, we asked whether a combination of powerful automated methods and in-depth manual analysis could reveal overlooked homologs of viral proteins classified as “genus restricted” by BLAST. To answer this question, we set up an automated pipeline that could run various sequence similarity detection methods and analyze the taxonomic distribution of the homologs they identified.

In the second part, we applied these methods to the genome of a phylogenetically isolated virus, Chronic bee paralysis virus (CBPV), a pathogen of the honeybee, in which most of the open reading frames (ORFs) were classified as orphans (20). We could find homologs of most of these ORFs and suggest putative functions for them. In particular, our results suggest that several insect and plant viruses that have different morphologies nevertheless have homologous structural proteins (SPs).

## MATERIALS AND METHODS

### Databases used.

We ran BLAST (11) and PSI-BLAST (11) against the NCBI nonredundant (nr) database (1 April 2012 release). We ran HHsearch searches against version 26 of PFAM (21) and HHblits against its own database of UniProt sequence clusters, UniProt20 (22) (2 December 2011 release). We relied on the NCBI taxonomy (2/3 April 2012 release) to map UniProt identifiers (from the UniProt 21 March 2012 release) to the NCBI taxonomy.

### Sequence similarity searches.

For homology searches, we ran BLAST, PSI-BLAST, HHblits, and HHsearch with the following parameters: BLAST, executable blastall, version 2.2.23, E value cutoff of 10^−3^, SEG low-complexity filtering enabled; PSI-BLAST, executable psiblast, version 2.2.26+, E value cutoff of 10^−3^, low-complexity filtering enabled, 10 iterations maximum; HHblits and HHsearch, executable hhblits and hhsearch from HHsuite, version 2.0.13, E value of 10^−3^, realignment using local maximum-accuracy algorithm enabled with a default maximum accuracy threshold parameter value of 0.35, four iterations maximum. We used the A3M multiple-sequence alignment generated by HHblits to run HHsearch.

### ANNOTATOR environment.

We used the ANNOTATOR web application (23) to run the search algorithms and calculate the raw taxonomy statistics. ANNOTATOR provides a convenient interface for running a comprehensive array of sequence analysis algorithms focused on protein function discovery. It is available at http://annotator.bii.a-star.edu.sg. The results were analyzed and tabulated by using a set of custom Perl scripts.

### Constitution of a data set of viral proteins classified by BLAST as genus restricted.

We adapted a data set of viral “ORFan” genes (species restricted) generously provided by YanBin Yin (3). To keep the size of the data set manageable, we focused on a subset composed of proteins from viruses with positive, single-stranded RNA viral genomes and for which BLAST detected no homologs in other genera by using the parameters and database described above. For the final data set, containing 351 sequences, see Table S1 in the supplemental material.

### Identifying the taxonomic distribution of homologs.

For each query, we compiled the taxonomic distribution of the hits retrieved by the similarity detection tools as follows. First, we collected all of the hits that had statistically significant similarity (E values of ≤10^−3^) to the query. For BLAST and PSI-BLAST, these hits correspond to single sequences and we retrieved their NCBI taxonomy. For HHblits, the hits correspond to clusters of protein sequences and we retrieved the NCBI taxonomy of all of the sequences listed in the clusters. For HHpred searches, the hits are “families” of protein sequences and we retrieved the taxonomy of all of the sequences from these families. Some PFAM families are grouped into “clans” (24). We retrieved the taxonomic distribution of all of the sequences from these clans. We discarded any nonviral sequence, which would have complicated the analysis without any benefit for our study (see Discussion). Second, having collected the taxonomic distribution of hits for each software, we counted the distinct taxons that were retrieved at the species, genus, and family ranks.

In some rare cases, virus species have no assigned genus or family. In these cases, we adjusted the taxonomic counts so that the counts for a particular query at a lower rank would be at least equal to the counts at a higher rank. For example, a query that had homologs in four viral families would also be counted as having homologs in four viral genera—even if the viral families in question did not have defined genera.

### In-depth manual homology detection incorporating contextual information.

To identify remote homologs missed by automated searches, we exploited “contextual” information, such as taxonomy, genome organization, and domain organization (25–28). Our procedure is similar to that described previously (29) and consists of two steps, the detection of potential homologs and their validation. We first identified “straightforward” homologs of the query protein in the NCBI nr database (1 April 2012 release) by using HHpred (14), HHblits (15), and CSI-BLAST (30, 31) and selecting hits whose E values were below the cutoff of 10^−3^. We then examined subsignificant hits (i.e., those with E values of >10^−3^) up to an E value of 2,000, looking for viral proteins or domains that came from a virus taxonomically related to the query (or infecting similar hosts) and/or that occurred in the same position of the genome or of the viral polyprotein. Such subsignificant hits, which have weak similarity to the query protein and occur in a similar genomic context, constitute potential homologs. To validate these candidates, we gathered homologs of these subsignificant hits (as described above, i.e., with E values of ≤10^−3^) and used HHalign (32) to compare homologs of the query protein (obtained as described above) with homologs of the subsignificant hits. We considered an HHalign E value of <10^−5^ to indicate homology between the subsignificant hit and the query. We performed additional checks, such as verifying that the secondary structure and function of the hits were compatible with those of the query. When we validated a potential homolog, we repeated the procedure after including it in query alignments (i.e., we performed iterative or “cascade” searches [33–35] until no new homologs were found).

### Detection of homologs of CBPV ORFs.

To detect homologs of ORF1, we obtained the following tools from their web servers and used them with default parameters: HHpred (14) (http://toolkit.tuebingen.mpg.de/hhpred), FFAS (1) (http://ffas.sanfordburnham.org/ffas-cgi/cgi/ffas.pl), and WebPRC (16) (http://www.ibi.vu.nl/programs/prcwww). We used PROMALS (36) to compare the secondary structure of ORF1 with that of known methyltranferase-guanylyltransferases (MTase-GTases).

We used contextual information coupled with sequence similarity searches as described above to detect homologs of ORF2 and ORF3. We obtained CSI-BLAST (30, 31) from its web server (http://toolkit.tuebingen.mpg.de/cs_blast#) and used it with five iterations and most of the default parameters (inclusion cutoff E value of 10^−3^, low-complexity filter not enabled, nr database). However, to examine as many subsignificant hits as possible, even extremely weak ones, we set the maximum E value reported to 2,000 (the default value is 10) and the maximum number of sequences reported to 2,000 (the default is 100).

We used ANNIE (37; http://annie.bii.a-star.edu.sg) to predict the structural properties of all of the ORFs of CBPV; MetaPrDOS (38) for disorder prediction, respecting the principles described in reference 39; Composition Profiler (40) for sequence composition analyses; and LOMETS (41) for fold recognition.

### Prediction of TM segments in CBPV ORF2 and ORF3.

To predict the number and locations of transmembrane (TM) segments of ORF2 and ORF3, we relied on two complementary approaches. On the one hand, for each virus, we compared the predictions of multiple programs for a single sequence (vertical approach). We considered the robustness of the prediction to be proportional to the number of predictors that detected a given TM segment. The predictors used were those applied and displayed by ANNIE (37). On the other hand, we compared the prediction of a single program for several homologs (horizontal approach) by using TM-coffee (42), which predicts TM segments in multiple sequences via HMMTOP (43). We considered the robustness of the prediction to be proportional to the number of sequences that contained a given predicted TM segment.

Finally, we used Phobius (44) to predict signal peptides and the topology of membrane proteins and TMSOC (45) to distinguish “complex” from “simple” anchor-type TM segments liable to give spurious hits in similarity searches.

## RESULTS

An intuitive understanding of the power of similarity search methods can be provided by the genetic distance at which they can detect homologs. However, the genetic distances between viral proteins often cannot be easily established by standard phylogenetic analyses, owing to their high rate of evolution (particularly in RNA viruses). Instead, we relied on the precomputed viral taxonomy as a proxy for genetic distances, since recent studies suggest that viral taxonomy reflects sequence-based phylogeny well (46–48) (despite being based on additional factors such as antigenicity).

We compiled a data set of 351 proteins from positive-sense, single-stranded RNA viruses that BLAST classifies as genus restricted by adapting a previously published data set identified as ORFans on the basis of BLAST searches (3) (see Table S1 in the supplemental material).

### Profile-profile methods find more distant homologs of 25% of the proteins classified as genus restricted by BLAST.

We first ran several automated sequence similarity search programs on this data set and compared the taxonomic depths at which they detected homologs. For a given program, a protein was deemed homologous to the query if the program reported a statistically significant sequence similarity between them, i.e., with an E value smaller than the cutoff of 10^−3^. The programs we compared included the widely used BLAST and PSI-BLAST programs and the more recent profile-profile comparison methods HHpred (14) and HHblits (15).

**FIG 1:**
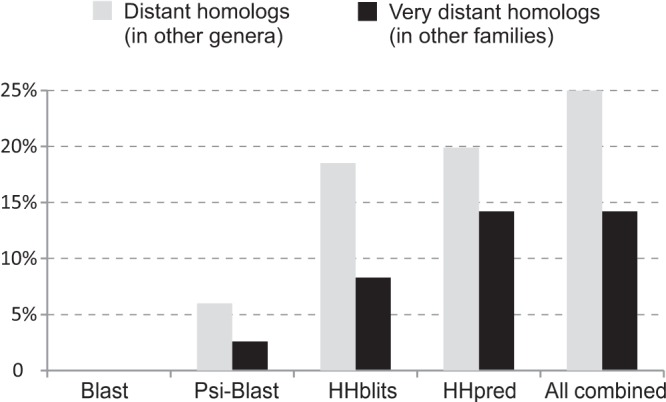
Capacities of the methods tested to detect homologs at different taxonomic depths. Shown are the proportions of proteins classified as genus restricted by BLAST and found by the different similarity search methods to have homologs beyond the genus level and beyond the family level. Precise values are in Table 1, columns 2 and 3.

If the proteins in the data set were truly genus restricted, then no algorithm would detect homologs in other genera. This is not what we observed. Table 1 and Fig. 1 show that all of the methods, aside from BLAST, detect homologs in other genera for at least some of the proteins in the data set. For example, HHpred detected distant homologs (i.e., in more than one genus) for about 20% of the sequences and very distant homologs (i.e., in more than one family) for about 14% of the sequences. Methods based on profile-profile comparison, i.e., HHpred and HHblits, detected many more distant homologs than PSI-BLAST (Fig. 1 and Table 1). Among these methods, HHpred found about as many distant homologs as HHblits but markedly more very distant homologs. This is probably due to the fact that contrary to HHblits, HHpred relies on a database of protein profiles built with human supervision (PFAM); in particular, PFAM clans often incorporate other information than merely sequence data, such as 3D structure or function (24). When combining results from PSI-BLAST, HHblits, and HHpred (right side of Fig. 1), in total, >25% of the 89 proteins in the data set had distant homologs and >14% had very distant homologs. Thus, many of the proteins characterized as genus restricted by BLAST and thought to be ORFans (3) are actually members of protein families with a wide taxonomic distribution.

**TABLE 1:** Capacities of the methods tested to detect homologs at different taxonomic depths^*a*^

Algorithm	% of sequences for which homologs were found with the following taxonomic distribution:		
	At most 1 genus	>1 genus	>1 family
BLAST	100	0	0
PSI-BLAST	94	6	2.6
HHblits	81.5	18.5	8.3
HHpred	80.1	19.9	14.2
All combined	74.6	25.4	14.2

a The total for each row can be >100% because “beyond genus level” includes “beyond family level.” Likewise, the total proportion of the different algorithms in each column can be greater than the value of the cell “all methods combined” because for some proteins, distant homologs were detected by several algorithms. Percentages were calculated from a total of 351 sequences.

For the proteins for which homologs were detected in more than one genus by at least one program and their taxonomic distribution according to each method, see Table S2 in the supplemental material. To corroborate these results, we examined in-depth 18 proteins (one-fifth) selected at random from among the 89 proteins found to have distant homologs (see Materials and Methods). We found that all were correct (not shown). We also ran the same calculations with a more stringent E value cutoff (10^−5^) and obtained qualitatively similar results (not shown), confirming that they are not an artifact due to the detection of false positives.

### Manual analysis using biological context reveals markedly more distant homologs than automated methods.

Even the profile-profile methods described above could not find distant homologs of 75% of the proteins in the data set with the standard significance cutoff (E = 10^−3^). However, more distant homologs can sometimes be detected by examining subsignificant hits (i.e., those with E values of >10^−3^) and using “extrinsic” or “contextual” sequence-based information that goes beyond simple sequence similarity (such as taxonomy, gene order, or domain organization) (25). We randomly selected 10 such proteins (Table 2) and analyzed them in depth manually (see Materials and Methods). This allowed the detection of more homologs in half of the cases (5 out of 10) listed in the top half of Table 2 (compare columns 4 and 5). The evidence supporting homology is described in the last column of Table 2.

**TABLE 2:** In-depth analysis, using contextual information, of a random subset of proteins classified as genus restricted by all automated methods

Accession no.	Protein name	Taxonomic position (family, genus, species)	Taxonomic distribution found by automated methods	Taxonomic distribution found by in-depth manual analysis	Type of protein (reference)	Evidence
YP_227375	Large coat protein	Secoviridae, unclassified, Strawberry latent ringspot virus	2 species	>40 families	Contains two domains of capsids with a jellyroll fold (PFAM clan Viral_ssRNA_CP) (49)	Marginal HHpred hits (E = 0.28 for region 241–308 and E = 7.5 for 43–159 region) to PFAM family RhV, HHalign comparison with RhV alignment (E = 5 × 10^−2^), functional confirmation (50)
NP_042511	Coat protein	Barnaviridae, Barnavirus, Mushroom bacilliform virus	1 species	>40 families	Capsid with a jellyroll fold (PFAM clan Viral_ssRNA_CP) (49)	Marginal HHpred hit (E = 0.2) to PFAM family Viral_Coat for 67–181 region, HHalign comparison with Viral_coat (E = 1.4 × 10^−3^), functional confirmation (51)
YP_308882	6K2	Potyviridae, Ipomovirus, Cucumber vein yellowing virus	4 species	4 genera in Potyviridae family (Ipomovirus, Poacevirus, Tritimovirus, Macluravirus)	6K2 (87)	Subsignificant CSI-BLAST hits to other 6K2 proteins (also located between CI protein and VPg protein), significant HHalign comparison between full-length 6K2 of Ipomovirus and 6K2 of Tritimovirus, Poacevirus, and Macluravirus (E = 2.9 × 10^−7^)
NP_776026	Putative matrix protein M	Flaviviridae, Flavivirus, Tamana bat virus	1 species	At least 1 whole genus (Flavivirus), may also be homologous to matrix protein of related genus Pegivirus	Membrane protein (52)	Subsignificant CSI-BLAST hits of M proteins of flaviviruses to Tamana bat virus M protein, which has identical domain position within polyprotein (between C and E proteins), significant HHalign comparison between 53–159 region of M of Tamana bat virus and M of other flaviviruses (E = 6 × 10^−6^)
NP_778215	VPg	Unclassified, Sobemovirus, Turnip rosette virus	1 species	1 whole genus (Sobemovirus)	Genome-linked protein Vpg (88)	CSI-BLAST finds full-length significant matches to VPg of many sobemoviruses, further iterative sequence searches identify as homologs VPg of all other sobemoviruses, all have same position within 2a/2b polyprotein (downstream of serine protease domain)
YP_293702	P9	Closteroviridae, Crinivirus, Tomato chlorosis virus	1 genus	1 genus	P9 (53)	
NP_619694	Hypothetical protein p34	Closteroviridae, Crinivirus, Lettuce infectious yellows virus	1 species	2 species^*a*^	Endonuclease	HHpred hit to RNase Dicer^*a*^ (PDB^*b*^ accession no. 3c4b, E = 2.10^−4^)
NP_851570	p22	Closteroviridae, Crinivirus, Cucurbit yellow stunting disorder virus	1 species	1 species		
YP_293697	p5	Closteroviridae, Crinivirus, Tomato chlorosis virus	1 species	1 species		
YP_053926	Hypothetical peptide	Comoviridae, Nepovirus (subgroup A), Tobacco ringspot virus	1 species	1 species		

a p34 has homologs in only two viral species but is homologous to a vast family of RNases from cellular organisms and thus most probably originated by horizontal transfer, which is beyond the scope of this study (see Materials and Methods and Discussion).

b PDB, Protein Data Bank.

Of these five proteins, three have homologs in more than one genus, including two that have homologs in more than one family. The 6K2 protein of Cucumber vein yellowing virus is homologous to the 6K2 proteins of several genera in the Potyviridae family (Table 2). The coat protein of Strawberry latent ringspot virus and that of Mushroom bacilliform virus are each predicted to have a jellyroll fold (49) and to have homologs in >40 families (Table 2). Experiments confirmed that they function as a capsid (50, 51).

Intriguingly, four of the five other genus-restricted proteins belong to the same family, Closteroviridae. The fact that this family contains numerous proteins that lack identifiable orthologs has been noted previously (53).

In summary, 3 of 10 proteins that all of the automated methods found to be genus restricted actually have distant homologs detectable by manual sequence analysis. This value cannot be simply extrapolated to the whole data set because of the small size of the subset examined. Nevertheless, it is clear that numerous proteins classified as genus restricted by BLAST and initially thought to be ORFans (3) actually have more distant homologs. Therefore, methods relying on profile-profile comparison should be used in addition to BLAST and PSI-BLAST to annotate viral genomes.

### Case study: in-depth analysis of the genome of CBPV.

We applied a combination of automated profile-profile methods and manual examination of subsignificant hits to analyze the genome of a phylogenetically isolated virus, CBPV (proposed genus, Chroparavirus [P. Blanchard, personal communication]), that induces paralysis in the honeybee Apis mellifera (20). The first genome segment (RNA1) encodes three ORFs (all accession numbers are in Table 3). ORF1 and ORF3 are thought to give rise to a fusion protein, ORF1-ORF3, by a frameshift (20). ORF2 overlaps ORF1; its expression is unproven. PSI-BLAST detected significant similarity between ORF3 and viral RNA-dependent RNA polymerases (RdRPs) but could detect no homolog of ORF1 or ORF2 (20). The second genome segment (RNA2) of CBPV contains three ORFs (ORF1 to ORF3) for which PSI-BLAST could detect no homolog either (20).

**TABLE 3:** accession numbers of ORFs of chronic bee viruses and homologous ORFs

Genus and species or products and host species	Protein name	Accession no.
Chroparavirus^*a*^		
Chronic bee paralysis virus (CBPV)	ORF1 from RNA1	YP_001911136.1
	ORF2 from RNA2	YP_001911140.1
	ORF3 from RNA2 (SP24)	YP_001911141.1
Anopheline-associated C virus (AACV)	ORF1	Being submitted
	ORF2	Being submitted
Sinaivirus^*b*^		
Lake Sinai virus 1	ORF1	AEH26192.1
Lake Sinai virus 2	ORF1	AEH26187
Cilevirus		
Citrus Leprosis virus C	p24	ABC75826.1
Citrus leprosis virus cytoplasmic type 2	p24	AGE82891.1
Blunervirus,^*c*^ Blueberry necrotic ring blotch virus	p24	YP_004901704.1
Higrevirus, Hibiscus green spot virus	p23	AER13452.1
Negevirus (Negev group)		
Negev virus	ORF2	AFI24682.1
	ORF3	AFI24674.1
Ngewotan virus	ORF2	AFY98073.1
Ngewotan virus	ORF3	AFY98074.1
Piura virus	ORF2	AFI24679.1
Piura virus	ORF3	AFI24680.1
Loreto virus	ORF3	AFI24694.1
Loreto virus	ORF2	AFI24692.1
Negevirus (Santana group)		
Santana virus	ORF2	AFI24676.1
Dezidougou	ORF2	AFI24670.1
Cellular proteins		
Drosophila melanogaster^*d*^	IP15837p	ABC86319.1
Glossina morsitans^*d*^	Hypothetical nonconserved protein	ADD20599.1

a Proposed genus (P. Blanchard, personal communication).

b Proposed genus (this study).

c Proposed genus (89).

d May be an endogenous viral protein (see text).

### ORF1 of CBPV RNA1 is homologous to the Alphavirus MTase-GTase.

HHpred reported a statistically significant hit (E = 4.6 × 10^−4^) between aa 132 to 325 of RNA1 ORF1 and the first 231 aa of the PFAM family Vmethyltransf, corresponding to the MTase-GTase of the Alphavirus supergroup (54). Two recently discovered viruses infecting bees have an organization similar to that of CBPV, Lake Sinai virus 1 and Lake Sinai virus 2 (55). Their first genome segment is also composed of an ORF1 with significant similarity to that of CBPV, followed by an ORF encoding the RdRP. We aligned the ORF1 of CBPV and those of the Lake Sinai viruses and submitted the alignment to HHpred. HHpred reported a longer match between this alignment (corresponding to aa 131 to 338 of CBPV) and almost the entire Vmethyltransf domain, albeit with a lower E value (E = 0.002), marginally under the threshold of significance. The strictly conserved histidine of the MTase-GTase of the Alphavirus superfamily (54) is also conserved in the ORF1 of the bee viruses (aa 158 in CBPV ORF1). In addition, the predicted secondary-structure elements of the putative MTase-GTase of CBPV matched that of the Alphavirus supergroup (not shown). Thus, we conclude that the aa 131 to 338 region of CBPV ORF1 contains a domain homologous to the MTase-GTase of the Alphavirus superfamily. This prediction is coherent with the facts that the genome of CBPV is capped (20) and that the MTase-GTase is generally found at the N terminus of the viral replicase (54). Finally, we could find no homolog of RNA1 ORF2.

### ORF3 of CBPV RNA2 is a putative virion membrane protein found in various insect and plant viruses.

We could detect no homologs of ORF2 and ORF3 of RNA2 by using HHblits or HHpred, but these programs would not detect sequences deposited very recently, since they rely on databases that are not updated daily. We therefore used CSI-BLAST (an improved version of PSI-BLAST [30, 31]), which searches the up-to-date NCBI nr database. We present first the analysis of ORF3 and then that of ORF2.

**FIG 2:**
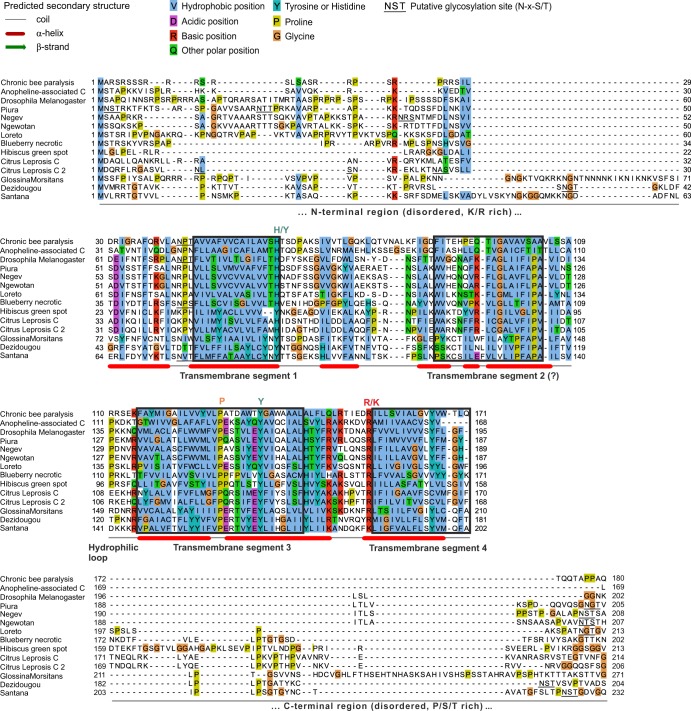
The SP24 family of virion membrane proteins of insect viruses. The boundaries of the predicted TM segments are approximative. We assumed that the topology of SP24 was conserved in all of the viruses, but in chroparaviruses (first two sequences), TM segment 2 is less hydrophobic and thus may be simply membrane associated, which would generate a different overall topology. The N-and C-terminal regions have no detectable sequence similarity and are presented only for information; whether they are homologous is unknown. Predicted N-glycosylation sites are indicated for N- and C-terminal regions only. Actual N-glycosylation can occur only if these regions are on the outside the virion, which we cannot reliably predict (see text).

CSI-BLAST on ORF3 from CBPV RNA2 detected protein p24 of Blueberry necrotic ring blotch virus (56) with a marginal E value (E = 0.13) but also a weaker similarity (E = 15) to ORF3 from Negev virus, the type species of Negevirus, a new genus of viruses infecting insects (57). Aligning these proteins with CBPV ORF3 and resubmitting the alignment to CSI-BLAST gave significant hits to several other viral or (apparently) cellular proteins (Table 3 and Fig. 2), i.e., p24 of Citrus Leprosis virus C (58–60), p23 of the related Hibiscus green spot virus (61), and proteins of insects. The latter probably corresponds to sequences of endogenous viruses integrated into Drosophila or Glossita genomes (62), since a tblastn search confirmed their presence in these genomes. To confirm the homology, we aligned ORF3 of CBPV with ORF3 of another recently discovered Chroparavirus, Anopheline-associated C virus (AACV; 90) and compared their alignment to that of the other proteins described above. The two groups had highly significant similarity (HHalign E value of 6 × 10^−14^) between the regions corresponding to aa 27 to 175 of CBPV ORF3 and aa 50 to 194 of Negev virus ORF3, confirming the homology. One cautionary note is that ORF3 and its homologs contain several TM segments (see below), which could give spurious hits in similarity searches if they were of a simple, anchor-like type (63). However, the program TMSOC (45) indicated that all of the TM segments of SP24 are complex, i.e., carry significant evolutionary information, and thus that the similarity is not spurious.

CBPV is the only virus for which functional information about ORF3 is available; it is thought to be an SP of the virion (20, 64, 65). We named ORF3 and its homologs SP24, owing to their size (20 to 24 kDa). Figure 2 presents an alignment of SP24 proteins. They are composed of an N-terminal region of 20 to 70 aa with low sequence complexity that is enriched in basic residues and predicted to be disordered in most species, a central region containing several predicted TM segments, and a C terminus predicted to be disordered and rich in P, S, and T. Therefore, SP24 is probably an integral membrane protein of the virion.

To predict the topology of SP24, we first tried to predict its number of TM segments and then to predict which region was inside or outside the virion. We used two complementary approaches to assess the robustness of TM segment predictions (see Materials and Methods). Overall, the predictions (data not shown) suggested that SP24 may contain four TM segments (Fig. 2). However, it is difficult to accurately predict TM segments in a multipass membrane protein, even when using consensus approaches (for instance, in a recent study of the NS2A protein of Dengue virus, a region predicted by all of the predictors to span the membrane was, in fact, found experimentally to be only membrane associated [66]), and therefore, only experiments can settle the matter.

Even assuming that there were four TM segments, we could not reliably predict which parts of SP24 were internal or external to the virion, since Phobius (44) gave discordant results for different homologs. Nevertheless, we made two observations. (i) The loop before TM segment 4 contains positively charged residues (R/K), and the very C terminus of SP24 contains potential N-glycosylation sites in negeviruses (Fig. 2). This suggests that the C terminus is on the outside of the virion. (ii) The N terminus of SP24 is basic, which would allow it to bind the viral RNA, if the N terminus were in the interior of the virion. Hypotheses i and ii are not simultaneously possible if there are four TM segments, since in that case both the N and C termini of SP24 would necessarily be on the same side of the membrane. However, it is possible that one of the four putative TM segments is, in fact, only membrane associated; a candidate would be segment 2, which is less hydrophobic, particularly in chroparaviruses (Fig. 2).

### ORF2 is probably a virion glycoprotein, detected in several insect viruses.

We next examined ORF2 of CBPV RNA2. CSI-BLAST reported a very weak hit (E = 691) to ORF2 of Piura virus, a Negevirus. This ORF2 has significant sequence similarity to the ORF2 of other negeviruses, except Santana virus and Dezidougou virus, which form a separate clade (we will call it the Santana group). A comparison of ORF2 of CBPV and AACV with ORF2 of negeviruses (with the Santana group excluded) confirmed that they were homologous (HHalign E value of 6 × 10^−7^ between the regions corresponding to aa 265 to 315 of CBPV ORF2 and aa 53 to 103 of Negev virus ORF3). We could not find other homologs of ORF2, even by in-depth examination of the genome of the insect viruses encoding SP24. Finally, we could not find homologs of the remaining ORF (ORF1) of CBPV RNA2.

**FIG 3:**
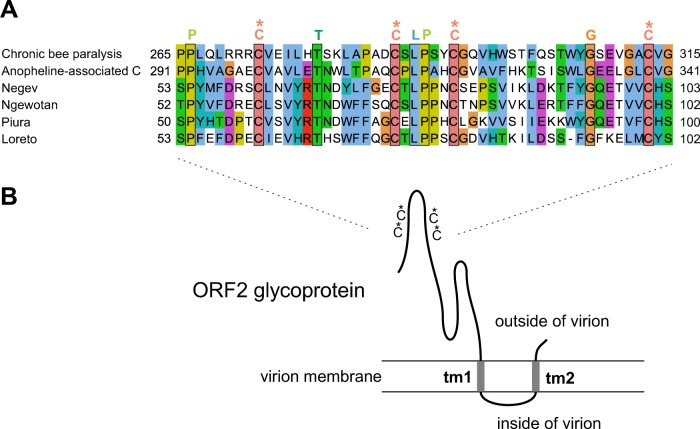
The viral ORF2 glycoproteins of chroparaviruses and negeviruses. (A) Alignment of the cysteine-rich region of ORF2 sequences of chroparaviruses and negeviruses. Conventions are the same as in Fig. 2. The conserved cysteines, predicted to form disulfide bridges, are indicated by an asterisk. (B) Predicted organization of ORF2. We make no claim to accurately predict disulfide connectivity. Other disulfide bridges are likely to occur elsewhere in ORF2 but are not conserved across taxons (see text). tm1 and tm2, TM segments 1 and 2, respectively.

The region of similarity between the ORF2 sequences of CBPV and negeviruses corresponds to 50 aa in the N-terminal or central part of ORF2, which contains nine conserved residues, including four cysteines (Fig. 3A), predicted to form disulfide bridges by Metaldetector (67). Outside of this region, we could detect no further sequence or secondary-structure similarity between ORF2 of CBPV and that of negeviruses. However, they are similar in organization, being composed of a predicted TM segment (or a signal peptide for negeviruses) 30 to 40 aa upstream of the conserved cysteine-rich region, followed by a variable region of about 200 aa, and two or three predicted C-terminal TM segments. In all of the viruses, ORF2 contains predicted N-glycosylation sites (not shown) and other cysteines conserved only in closely related species, which may form other disulfide bridges. Thus, ORF2 has all of the features of a virion glycoprotein. A speculative model of its topology is presented in Fig. 3B.

**FIG 4:**
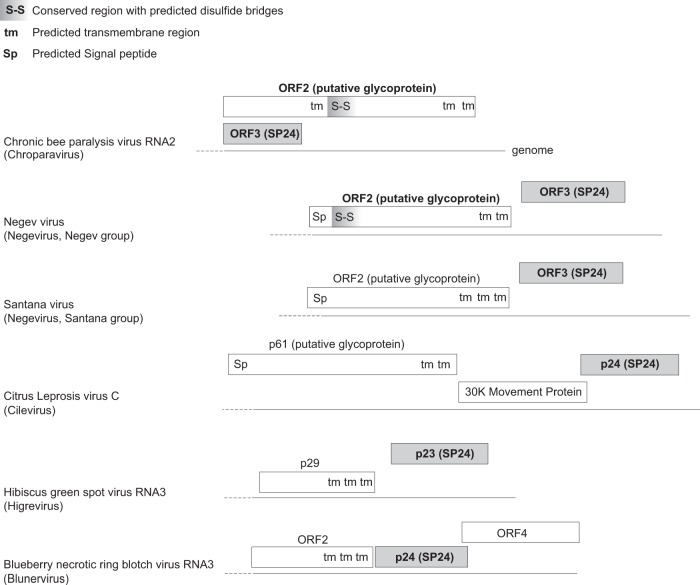
Comparison of SP24 and the ORF2 glycoprotein in different viruses. The genomic contexts of SP24 in insect viruses are shown. Genomes and proteins are approximately to scale. The names of proteins that have significant sequence similarity (and are thus demonstrably homologs) are in bold.

Figure 4 presents a summary of the organization of ORF2 and ORF3 and of their genomic context in different viruses. The long (200-aa) N-terminal extension of ORF2 (predicted to be disordered) that overlaps ORF3 only in chroparaviruses (Fig. 4, top) probably originated by overprinting (29, 68, 69) in their common ancestor. Since the predicted membrane segment of CBPV ORF2 occurs in the same position as the signal peptide of Negevirus ORF2 (compare the first two viruses in Fig. 4), it may be cleaved to give rise to the same topology.

Finally, we searched for putative glycoproteins analogous to ORF2 in other viruses that encode SP24. Cilevirus p61 and ORF2 of the Santana group of negeviruses have features similar to those of ORF2 and may be their functional equivalent (Fig. 4). Blunervirus and Higrevirus also encode proteins with predicted C-terminal TM segments (Fig. 4), but they are considerably shorter than ORF2 or p61.

### Putative roles of SP24 and ORF2 in light of experimental data.

The viruses that encode an SP24 homolog have a variety of morphologies (Table 4), spherical (Negevirus), ellipsoidal (Chroparavirus), and short, bacilliform (Cilevirus, Higrevirus). Interestingly, these three groups of morphologies correlate with the phylogeny of the viral replicative enzymes. Since CBPV SP24 and ORF2 are thought to be SPs (20, 64, 65), they are probably the main membrane virion proteins. The fact that SP24 and ORF2 are both also encoded by negeviruses suggests that they may interact. How they would underlie a different morphology in these viruses is unclear; we note, however, that treatment of CBPV virions with acids or bases gave rise to a nearly spherical morphology similar to that of negeviruses (65). It is unclear whether chroparaviruses and cileviruses have enveloped virions. Chroparaviruses are regularly cited as being nonenveloped (e.g., reference 70), though to the best of our knowledge, this is not firmly proven. Cileviruses are also regularly cited as being nonenveloped (e.g., reference 71), but earlier reports described them as enveloped (72, 73). Obviously, if our hypotheses regarding the role of SP24 are correct and if its function is conserved, then these viruses must be enveloped.

**TABLE 4:** virion morphology of viruses encoding an SP24 matrix protein

Genus	Type species	Morphology	Host(s)	Reference(s)
Chroparavirus (proposed)	Chronic bee paralysis virus	Ellipsoidal, different populations (220 by 41, 54, or 64 nm), treatment with acid or base solutions results in more rounded, apparently empty shells (20–30 by 20–50 nm)	Insects (bees, mosquitoes)	65
Cilevirus	Citrus Leprosis virus C	Short, membrane-bound, enveloped, bacilliform particles (40–50 by 80–120 nm)	Plants (citrus), insects (erythrophyte mites)	60, 72, 73
Higrevirus	Hibiscus green spot virus	Short, bacilliform particles (30 by 50 nm)	Plant (hibiscus), insects (erythrophyte mites)	61
Blunervirus (proposed)	Blueberry necrotic ring blotch virus	Unknown	Plant (blueberry), probably transmitted by erythrophyte mites	
Negevirus	Negev virus	Spherical, enveloped particles (45–55 nm)	Insects (mosquitoes, phlebotomine sand flies)	57

Most plant viruses encode a capsid protein that gives them a flexuous, icosahedral, or tubular morphology (74). Thus, SP24 is probably a new type of SP of plant and insect viruses. Its predicted topology is reminiscent of the Coronaviridae M protein, which contains three TM segments and a membrane-associated region (75) and forms spherical particles.

In conclusion, a combination of automated profile-profile methods and in-depth manual analysis allowed the detection of remote homologs and gave functional clues about most of the ORFs of a phylogenetically isolated virus.

## DISCUSSION

### Does the similarity among SP24, ORF2, and other viral proteins come from homology?

Significant sequence similarity is widely considered evidence of homology, because there is no imperious constraint on protein sequences that would make convergent evolution likely (i.e., very different sequences can perform the same function or adopt the same structure [76]). However, this is not rigorously applicable to regions with low sequence complexity, since E values have been calibrated on globular proteins. In addition, convergent evolution should always be considered if there is no plausible mechanism by which two proteins could have evolved by common descent.

Several lines of argument strongly suggest that the similarity among SP24, ORF2, and other viral proteins is due to homologous descent. (i) The region similar among ORF2 proteins is most likely globular (Fig. 3), and thus, the caveat above does not apply. (ii) The TM segments of SP24 are predicted to carry significant evolutionary information, unlike simple “anchor” TM segments (45). (iii) The fact that both ORF2 and ORF3 of chroparaviruses have significant similarity to those of negeviruses considerably strengthens the homology hypothesis, since it seems difficult to envision why convergent evolution would have occurred twice. (iv) There is a plausible mechanism to explain homology, i.e., horizontal transfer between similar organisms (RNA viruses) that infect similar hosts (insects and plants).

### Manual analyses incorporating contextual information are an indispensable complement of automated searches on viral proteins.

Our study shows that making use of the biological context and examining search results far beyond the threshold of statistical significance allows the detection of homologs even in phylogenetically isolated viruses. Such approaches are successful for three main reasons. (i) Gene and domain order are often conserved in viruses, at least within the same family. For instance, the order of domains is mostly conserved in the Flaviviridae polyprotein (77), as is the order of genes in the Coronaviridae genome (78). (ii) Because RNA viruses have very few genes, a weak hit to a protein from a related virus conveys strong information (contrary to, for instance, a weak hit from a human protein to another). (iii) Though it remains difficult to detect very distant homologs, it has recently become easier to validate candidate homologs by pairwise profile-profile comparison (32).

The results presented here suggest that the “limits of homology detection” (79) are far from having been reached for viral proteins, despite their fast evolution. In particular, proteins that have strictly conserved residues owing to catalytic activity (such as the presumed MTase-GTase of CBPV) are expected to retain detectable sequence similarity over long distances.

### A cautionary note: checks to perform when doing manual analyses.

Some well-established checks that will avoid many false positives in sequence similarity searches include (17, 39, 68, 80) (i) excluding regions with low sequence complexity, coiled coils, disordered regions (by using, for instance, ANNIE [37]), and simple TM segments (45); (ii) comparing the lengths of the query and the hit (viral proteins rarely change dramatically in length, unlike eukaryotic proteins, except in specific cases like polyproteins), their functions, and their secondary structures; (iii) using a relatively stringent cutoff for the validation step (e.g., HHalign E values of <10^−5^) of candidate homologs identified in the detection step; and (iv) waiting for the sequence of new, divergent viruses if there remains a doubt over a prediction; they will often settle the matter.

### Limitations of our study and comparison with previous studies.

A limitation of our work is that we only considered viral homologs of the proteins of the ORFan data set, because cellular homologs often correspond to horizontal transfer (81), for instance, isolated cases of endogenous viruses (82, 83). The limitations of BLAST have already been noted in archaeal viruses (84) and mimiviruses (85).

### Implications for the study of ORFans.

In conclusion, we suspect that our results are applicable to all organisms and not only viruses. BLAST can reliably identify ORFans in mammalian genomes (86) owing to their low rate of evolution. However, as the phylogenetic distance increases (for instance, when comparing vertebrates and invertebrates), homologs are expected to be increasingly difficult to detect. Tautz et al. wrote in a recent review “we are still missing a systematic study that uses PSI-BLAST-based searches to provide a reliable estimate of orphan gene affiliation to the known protein folds” (9). We agree but suggest that more powerful tools should also be used, such as profile-profile comparison or fold recognition.

## Supplementary Material

Supplemental material
