# Supplementary material for: Powerful Sequence Similarity Search Methods and In-Depth Manual Analyses Can Identify Remote Homologs in Many Apparently “Orphan” Viral Proteins
Source: J Virol. 2014 Jan;88(1):10–20. doi: 10.1128/JVI.02595-13 (PMC3911697; doi:10.1128/JVI.02595-13)
Supplement: Supplemental material [file supp_88_1_10__index.html]

Powerful Sequence Similarity Search Methods and In-Depth Manual Analyses Can Identify Remote Homologs in Many Apparently “Orphan” Viral Proteins — Supplemental material 

# Powerful Sequence Similarity Search Methods and In-Depth Manual Analyses Can Identify Remote Homologs in Many Apparently “Orphan” Viral Proteins

## Supplemental material

**Files in this Data Supplement:**

- Supplemental file 1 -

  Table S1 (Data set of viral orphans.)

  Table S2 (Taxonomic distribution of proteins for which at least one method finds homologs in more than one genus.)

  PDF, 83K
