# Supplementary material for: Powerful Sequence Similarity Search Methods and In-Depth Manual Analyses Can Identify Remote Homologs in Many Apparently “Orphan” Viral Proteins
Source: J Virol. 2014 Jan;88(1):10–20. doi: 10.1128/JVI.02595-13 (PMC3911697; doi:10.1128/JVI.02595-13)
Supplement: Supplemental material [file JVI.02595-13_zjv999098468so1.pdf]

Supplemental Material (2 Tables)

Supplementary Table S1: dataset of viral orphans

Dataset of 351 positive-strand, single-stranded (+ssRNA) viral “orphan” proteins for which BLAST was unable to find homologues in other viral genera. The dataset was adapted from a larger dataset generously provided by Yanbin Yin (see main text for reference and Materials and Methods). The protein names and taxonomy listed in the table were taken directly from this dataset.

Accession	Description	Taxonomy
NP_065656	glycoprotein 2b (GP2b) [Equine arteritis virus]	Nidovirales; Arteriviridae; Arterivirus
NP_065657	glycoprotein 3 (GP3) [Equine arteritis virus]	Nidovirales; Arteriviridae; Arterivirus
NP_065658	glycoprotein 4 (GP4) [Equine arteritis virus]	Nidovirales; Arteriviridae; Arterivirus
NP_065659	glycoprotein 5 (GP5) [Equine arteritis virus]	Nidovirales; Arteriviridae; Arterivirus
NP_065660	hypothetical protein [Equine arteritis virus]	Nidovirales; Arteriviridae; Arterivirus
NP_065661	hypothetical protein [Equine arteritis virus]	Nidovirales; Arteriviridae; Arterivirus
NP_068550	coat protein [Botrytis virus F]	unclassified ssRNA positive-strand viruses
NP_077082	'8 KDa' triple gene block protein [Clover yellow mosaic virus]	Flexiviridae; Potexvirus
NP_085469	lysis protein [Bacteriophage AP205]	Leviviridae; Levivirus; unclassified Levivirus
NP_085472	coat protein [Bacteriophage AP205]	Leviviridae; Levivirus; unclassified Levivirus
NP_112032	triple gene block protein 4 [Banana mild mosaic virus]	Flexiviridae; unassigned species in Flexiviridae
NP_114360	putative protein P0 [Beet chlorosis virus]	Luteoviridae; Polerovirus
NP_115455	putative movement protein [Maize rayado fino virus]	Tymoviridae; Marafivirus
NP_127508	envelope (E) protein [Equine arteritis virus]	Nidovirales; Arteriviridae; Arterivirus
NP_148779	15 kDa protein [Cactus virus X]	Flexiviridae; Potexvirus
NP_148780	8 kDa protein [Cactus virus X]	Flexiviridae; Potexvirus
NP_150078	4.9 kDa non-structural protein [Bovine coronavirus]	Nidovirales; Coronaviridae; Coronavirus; Group 2 species
NP_150079	4.8 kDa non-structural protein [Bovine coronavirus]	Nidovirales; Coronaviridae; Coronavirus; Group 2 species
NP_203544	ORF2 [Simian hemorrhagic fever virus]	Nidovirales; Arteriviridae; Arterivirus
NP_203545	ORF2a [Simian hemorrhagic fever virus]	Nidovirales; Arteriviridae; Arterivirus
NP_203546	ORF3 [Simian hemorrhagic fever virus]	Nidovirales; Arteriviridae; Arterivirus
NP_203547	ORF4 [Simian hemorrhagic fever virus]	Nidovirales; Arteriviridae; Arterivirus
NP_203548	ORF5 [Simian hemorrhagic fever virus]	Nidovirales; Arteriviridae; Arterivirus
NP_203549	ORF6 [Simian hemorrhagic fever virus]	Nidovirales; Arteriviridae; Arterivirus
NP_203552	capsid protein [Simian hemorrhagic fever virus]	Nidovirales; Arteriviridae; Arterivirus
NP_203556	6.4 kDa triple gene block protein [Indian citrus ringspot virus]	Flexiviridae; Mandarivirus
NP_203558	putative 23 kDa nucleic acid binding protein [Indian citrus ringspot virus]	Flexiviridae; Mandarivirus
NP_573541	putative replicase [Euprosterna elaeasa virus]	Tetraviridae; Betatetravirus
NP_597749	Charged protein [Tobacco mosaic virus]	Tobamovirus
NP_604482	gamma B protein [Barley stripe mosaic virus]	Hordeivirus
NP_608313	7 kDa protein [Tobacco necrosis virus D]	Tombusviridae; Necrovirus
NP_608315	7 kDa protein [Tobacco necrosis virus D]	Tombusviridae; Necrovirus
NP_612591	8KDa protein [beet soil-borne mosaic virus]	Benyvirus
NP_613256	p11K protein [Carnation ringspot virus]	Tombusviridae; Dianthovirus
NP_613284	hypothetical protein [Cowpea mosaic virus]	Comoviridae; Comovirus
NP_613269	movement protein [Cowpea mottle virus]	Tombusviridae; Carmovirus
NP_613270	movement protein [Cowpea mottle virus]	Tombusviridae; Carmovirus
NP_613272	hypothetical protein [Cowpea mottle virus]	Tombusviridae; Carmovirus
NP_619562	TGB3 protein [Shallot latent virus]	Flexiviridae; Carlavirus
NP_619694	hypothetical protein [Lettuce infectious yellows virus]	Closteroviridae; Crinivirus
NP_619699	unknown [Lettuce infectious yellows virus]	Closteroviridae; Crinivirus
NP_619663	unknown [Grapevine virus A]	Flexiviridae; Vitivirus
NP_619666	putative RNA binding protein [Grapevine virus A]	Flexiviridae; Vitivirus
NP_619655	ORF2 [Grapevine virus B]	Flexiviridae; Vitivirus
NP_619673	hypothetical protein [Hibiscus chlorotic ringspot virus]	Tombusviridae; Carmovirus
NP_619674	putative movement protein P8 [Hibiscus chlorotic ringspot virus]	Tombusviridae; Carmovirus

NP_619675	putative movement protein P9 [Hibiscus chlorotic ringspot virus]	Tombusviridae; Carmovirus
NP_619677	hypothetical protein [Hibiscus chlorotic ringspot virus]	Tombusviridae; Carmovirus
NP_619717	hypothetical protein [Maize chlorotic mottle virus]	Tombusviridae; Machlomovirus
NP_619746	8 kDa protein [Potato aucuba mosaic virus]	Flexiviridae; Potexvirus
NP_619749	8 kDa protein [Potato aucuba mosaic virus]	Flexiviridae; Potexvirus
NP_619773	coat protein [Pelargonium zonate spot virus]	Bromoviridae
NP_619754	hypothetical protein [Oat chlorotic stunt virus]	Tombusviridae; Avenavirus
NP_619735	hypothetical 34K protein [Pea enation mosaic virus-1]	Luteoviridae; Enamovirus
NP_620039	capsid protein [Olive latent virus 2]	Bromoviridae; Oleavirus
NP_620110	protein B2 [Pariacoto virus]	Nodaviridae; Alphanodavirus
NP_620441	cysteine rich protein [Potato mop-top virus]	Pomovirus
NP_620466	movement protein [Raspberry bushy dwarf virus]	Idaeovirus
NP_620467	coat protein [Raspberry bushy dwarf virus]	Idaeovirus
NP_620503	P1 [Ryegrass mottle virus]	Sobemovirus
NP_620558	protein P2 [Acyrthosiphon pisum virus]	unclassified ssRNA positive-strand viruses
NP_620645	8K triple gene block protein [Strawberry mild yellow edge virus]	Flexiviridae; Potexvirus
NP_620647	11K hypothetical protein [Strawberry mild yellow edge virus]	Flexiviridae; Potexvirus
NP_620683	hypothetical protein TRVs2gp2 [Tobacco rattle virus]	Tobravirus
NP_620684	hypothetical protein TRVs2gp3 [Tobacco rattle virus]	Tobravirus
NP_620722	P8 protein [Turnip crinkle virus]	Tombusviridae; Carmovirus
NP_620718	triple gene block protein 3 [White clover mosaic virus]	Flexiviridae; Potexvirus
NP_620839	13K triple gene block protein [Plantago asiatica mosaic virus]	Flexiviridae; Potexvirus
NP_624334	movement protein [Citrus leaf blotch virus]	Flexiviridae; unassigned species in Flexiviridae
NP_624316	movement protein 3 [Sugarcane striate mosaic associated virus]	Flexiviridae; unassigned species in Flexiviridae
NP_660173	ORF 4 [Beet western yellows ST9 associated virus]	unclassified ssRNA positive-strand viruses
NP_663727	TGB3 protein [Pepino mosaic virus]	Flexiviridae; Potexvirus
NP_689397	putative ribonuclease III [Sweet potato chlorotic stunt virus]	Closteroviridae; Crinivirus
NP_689398	putative membrane protein [Sweet potato chlorotic stunt virus]	Closteroviridae; Crinivirus
NP_689399	hypothetical protein [Sweet potato chlorotic stunt virus]	Closteroviridae; Crinivirus
NP_689400	p6 protein [Sweet potato chlorotic stunt virus]	Closteroviridae; Crinivirus
NP_689403	hypothetical protein [Sweet potato chlorotic stunt virus]	Closteroviridae; Crinivirus
NP_702991	triple gene block3 [Tulip virus X]	Flexiviridae; Potexvirus
NP_715626	hypothetical protein SCMoVgp1 [Subterranean clover mottle virus]	Sobemovirus
NP_740767	6KDa glycine-rich protein [Broad bean necrosis virus]	Pomovirus
NP_778214	unknown [Turnip rosette virus]	Sobemovirus
NP_778215	unknown [Turnip rosette virus]	Sobemovirus
NP_783208	coat protein [Oyster mushroom spherical virus]	unclassified ssRNA positive-strand viruses
NP_783204	12 kDa unknown protein [Oyster mushroom spherical virus]	unclassified ssRNA positive-strand viruses
NP_783205	12.5 kDa unknown protein [Oyster mushroom spherical virus]	unclassified ssRNA positive-strand viruses
NP_783206	21 kDa unknown protein [Oyster mushroom spherical virus]	unclassified ssRNA positive-strand viruses
NP_783207	14.5 kDa unknown protein [Oyster mushroom spherical virus]	unclassified ssRNA positive-strand viruses
NP_783209	23 kDa unknown protein [Oyster mushroom spherical virus]	unclassified ssRNA positive-strand viruses
NP_803170	protein F [Hepatitis C virus]	Flaviviridae; Hepacivirus
NP_813797	6 kDa protein [Grapevine leafroll-associated virus 3]	Closteroviridae; Ampelovirus
NP_813800	55 kDa protein [Grapevine leafroll-associated virus 3]	Closteroviridae; Ampelovirus
NP_813803	21 kDa protein [Grapevine leafroll-associated virus 3]	Closteroviridae; Ampelovirus
NP_813804	19.6 kDa protein [Grapevine leafroll-associated virus 3]	Closteroviridae; Ampelovirus
NP_813805	19.7 kDa protein [Grapevine leafroll-associated virus 3]	Closteroviridae; Ampelovirus
NP_813807	7 kDa protein [Grapevine leafroll-associated virus 3]	Closteroviridae; Ampelovirus
NP_828853	hypothetical protein sars3b [SARS coronavirus]	Nidovirales; Coronaviridae; Coronavirus
NP_828854	protein E [SARS coronavirus]	Nidovirales; Coronaviridae; Coronavirus
NP_828856	hypothetical protein sars6 [SARS coronavirus]	Nidovirales; Coronaviridae; Coronavirus
NP_828857	hypothetical protein sars7a [SARS coronavirus]	Nidovirales; Coronaviridae; Coronavirus
NP_828859	hypothetical protein sars9b [SARS coronavirus]	Nidovirales; Coronaviridae; Coronavirus
NP_835246	p6 protein [Grapevine rootstock stem lesion associated virus]	Closteroviridae; Closterovirus; unclassified Closterovirus
NP_835251	p19 protein [Grapevine rootstock stem lesion associated virus]	Closteroviridae; Closterovirus; unclassified Closterovirus
NP_835252	p24 protein [Grapevine rootstock stem lesion associated virus]	Closteroviridae; Closterovirus; unclassified Closterovirus
NP_828852	hypothetical protein sars3a [SARS coronavirus]	Nidovirales; Coronaviridae; Coronavirus
NP_840018	P6 protein [Barley yellow dwarf virus - PAV]	Luteoviridae; Luteovirus
NP_840019	hypothetical protein [Barley yellow dwarf virus - PAV]	Luteoviridae; Luteovirus
NP_849176	hypothetical protein sars8a [SARS coronavirus]	Nidovirales; Coronaviridae; Coronavirus
NP_849177	hypothetical protein sars8b [SARS coronavirus]	Nidovirales; Coronaviridae; Coronavirus
NP_851568	p5.2 protein [Cucurbit yellow stunting disorder virus]	Closteroviridae; Crinivirus

NP_851569	p25 protein [Cucurbit yellow stunting disorder virus]	Closteroviridae; Crinivirus
NP_851570	p22 protein [Cucurbit yellow stunting disorder virus]	Closteroviridae; Crinivirus
NP_851573	p6 [Cucurbit yellow stunting disorder virus]	Closteroviridae; Crinivirus
NP_851575	p9 [Cucurbit yellow stunting disorder virus]	Closteroviridae; Crinivirus
NP_862838	p6 product [Pea stem necrosis virus]	Tombusviridae; Carmovirus
NP_891564	p14 [Little cherry virus 2]	Closteroviridae; Ampelovirus
NP_891566	p6 [Little cherry virus 2]	Closteroviridae; Ampelovirus
NP_891568	p53 [Little cherry virus 2]	Closteroviridae; Ampelovirus
NP_891569	p22 [Little cherry virus 2]	Closteroviridae; Ampelovirus
NP_891571	p26 [Little cherry virus 2]	Closteroviridae; Ampelovirus
NP_891561	p18 [Little cherry virus 2]	Closteroviridae; Ampelovirus
NP_919038	capsid protein [Macrobrachium rosenbergii nodavirus]	Nodaviridae; unclassified Nodaviridae
NP_932308	30kDa protein [Botrytis virus X]	unclassified ssRNA positive-strand viruses
NP_932310	14kDa protein [Botrytis virus X]	unclassified ssRNA positive-strand viruses
NP_932311	14kDa protein [Botrytis virus X]	unclassified ssRNA positive-strand viruses
NP_940786	ORF 2 [Beet pseudo-yellows virus]	Closteroviridae; Crinivirus
NP_940787	p9 [Beet pseudo-yellows virus]	Closteroviridae; Crinivirus
NP_940789	p6 [Beet pseudo-yellows virus]	Closteroviridae; Crinivirus
NP_941375	hypothetical protein CfMVp1 [Cocksfoot mottle virus]	Sobemovirus
NP_958174	triple block protein 3 [Poplar mosaic virus]	Flexiviridae; Carlavirus
NP_995578	hypothetical protein SMVAs1p2 [Sclerophthora macrospora virus A]	unclassified ssRNA positive-strand viruses
YP_025083	ORF 2 precursor [Strawberry pallidosis associated virus]	Closteroviridae; Crinivirus; unclassified Crinivirus
YP_025084	p8 [Strawberry pallidosis associated virus]	Closteroviridae; Crinivirus; unclassified Crinivirus
NP_758812	5K protein [Beet black scorch virus]	Tombusviridae; Necrovirus
YP_052930	6 kDa unknown protein [Pelargonium chlorotic ring pattern virus]	Tombusviridae; unclassified Tombusviridae
YP_054410	triple gene block protein 3 [Opuntia virus X]	Flexiviridae; Potexvirus; unclassified Potexvirus
YP_054412	hypothetical protein [Potato yellow vein virus]	Closteroviridae; Crinivirus; unclassified Crinivirus
YP_054416	putative hydrophobic membrane protein [Potato yellow vein virus]	Closteroviridae; Crinivirus; unclassified Crinivirus
YP_054418	hypothetical protein [Potato yellow vein virus]	Closteroviridae; Crinivirus; unclassified Crinivirus
YP_077185	p0 protein [Carrot red leaf virus]	Luteoviridae; unclassified Luteoviridae
YP_164803	unknown protein [Fragaria chiloensis latent virus]	Bromoviridae; Ilarvirus; Ilarvirus subgroup 6
YP_164806	ORF 3 [Fragaria chiloensis latent virus]	Bromoviridae; Ilarvirus; Ilarvirus subgroup 6
YP_164261	triple gene block protein 3 [Sweet potato chlorotic fleck virus]	Flexiviridae; Carlavirus; unclassified Carlavirus
YP_164263	nucleic acid binding protein [Sweet potato chlorotic fleck virus]	Flexiviridae; Carlavirus; unclassified Carlavirus
YP_224087	triple gene block protein [Hydrangea ringspot virus]	Flexiviridae; Potexvirus
YP_224089	virally coded protein [Hydrangea ringspot virus]	Flexiviridae; Potexvirus
YP_224092	p7 [Mint virus 1]	Closteroviridae; Closterovirus; unclassified Closterovirus
YP_224097	unknown [Mint virus 1]	Closteroviridae; Closterovirus; unclassified Closterovirus
YP_224137	TGB 3 [Mint virus X]	Flexiviridae; Potexvirus; unclassified Potexvirus
YP_224220	hypothetical protein CSDaVgp3 [Citrus sudden death-associated virus]	Tymoviridae; Marafivirus; unclassified Marafivirus
YP_227361	p6 [Blackberry yellow vein virus]	Closteroviridae; Crinivirus; unclassified Crinivirus
YP_238477	p13 [Pelargonium line pattern virus]	Tombusviridae; unclassified Tombusviridae
YP_263306	triple gene block protein 3 [Lily virus X]	Flexiviridae; Potexvirus
YP_277432	7K protein [Potato virus S]	Flexiviridae; Carlavirus
YP_293698	P4 [Tomato chlorosis virus]	Closteroviridae; Crinivirus
YP_293700	P8 [Tomato chlorosis virus]	Closteroviridae; Crinivirus
YP_293702	P9 [Tomato chlorosis virus]	Closteroviridae; Crinivirus
YP_293706	P7 [Tomato chlorosis virus]	Closteroviridae; Crinivirus
YP_293696	P22 [Tomato chlorosis virus]	Closteroviridae; Crinivirus
YP_293697	P5 [Tomato chlorosis virus]	Closteroviridae; Crinivirus
NP_040352	putative vector transmission protein [Pea early browning virus]	Tobravirus
NP_040353	putative vector transmission protein [Pea early browning virus]	Tobravirus
NP_056787	hypothetical protein HEVgp09 [Hepatitis E virus]	Hepeviridae; Hepevirus
NP_040783	10K hypothetical protein [Narcissus mosaic virus]	Flexiviridae; Potexvirus
NP_040832	3a protein [Avian infectious bronchitis virus]	Nidovirales; Coronaviridae; Coronavirus
NP_040833	3b protein [Avian infectious bronchitis virus]	Nidovirales; Coronaviridae; Coronavirus
NP_040834	small virion-associated protein [Avian infectious bronchitis virus]	Nidovirales; Coronaviridae; Coronavirus
NP_040836	5a protein [Avian infectious bronchitis virus]	Nidovirales; Coronaviridae; Coronavirus
NP_040837	5b protein [Avian infectious bronchitis virus]	Nidovirales; Coronaviridae; Coronavirus
NP_056756	8 kD membrane-bound protein [Potato virus X]	Flexiviridae; Potexvirus
NP_040991	6K triple gene block protein [Foxtail mosaic virus]	Flexiviridae; Potexvirus

NP_041195	coat protein [Alfalfa mosaic virus]	Bromoviridae; Alfamovirus
NP_041737	hypothetical protein [Rice yellow mottle virus]	Sobemovirus
NP_041871	putative membrane-binding protein [Beet yellows virus]	Closteroviridae; Closterovirus
NP_041876	long-distance transport factor [Beet yellows virus]	Closteroviridae; Closterovirus
NP_041877	RNA silencing suppressor [Beet yellows virus]	Closteroviridae; Closterovirus
NP_041886	p9 protein [Cardamine chlorotic fleck virus]	Tombusviridae; Carmovirus
NP_042300	hypothetical protein [Southern cowpea mosaic virus]	Sobemovirus
NP_042305	coat protein [Pseudomonas phage PP7]	Leviviridae; Levivirus; unclassified Levivirus
NP_042306	lysis protein [Pseudomonas phage PP7]	Leviviridae; Levivirus; unclassified Levivirus
NP_042508	orf1 [Mushroom bacilliform virus]	Barnaviridae; Barnavirus
NP_042511	coat protein [Mushroom bacilliform virus]	Barnaviridae; Barnavirus
NP_042583	hypothetical 14k protein [Bamboo mosaic virus]	Flexiviridae; Potexvirus
NP_042586	transmembrane protein [Bamboo mosaic virus]	Flexiviridae; Potexvirus
NP_042862	33-kDa protein [Citrus tristeza virus]	Closteroviridae; Closterovirus
NP_042863	6-kDa protein [Citrus tristeza virus]	Closteroviridae; Closterovirus
NP_042868	18-kDa protein [Citrus tristeza virus]	Closteroviridae; Closterovirus
NP_042869	13-kDa protein [Citrus tristeza virus]	Closteroviridae; Closterovirus
NP_043458	unknown [Lucerne transient streak virus]	Sobemovirus
NP_043459	unknown [Lucerne transient streak virus]	Sobemovirus
NP_054008	hypothetical protein [Carrot mottle mimic virus]	Umbravirus
NP_044333	triple gene block protein 3 [Papaya mosaic virus]	Flexiviridae; Potexvirus
NP_056829	unknown [Tobacco necrosis virus A]	Tombusviridae; Necrovirus
NP_044385	unknown1 [Saguaro cactus virus]	Tombusviridae; Carmovirus
NP_044389	unknown2 [Saguaro cactus virus]	Tombusviridae; Carmovirus
NP_054028	triple gene block 3 [Cymbidium mosaic virus]	Flexiviridae; Potexvirus
NP_044735	movement protein [Galinsoga mosaic virus]	Tombusviridae; Carmovirus
NP_044743	ORF 4 [Leek white stripe virus]	Tombusviridae; Necrovirus
NP_045003	hypothetical protein [Little cherry virus 1]	Closteroviridae; unclassified Closteroviridae
NP_045008	hypothetical protein [Little cherry virus 1]	Closteroviridae; unclassified Closteroviridae
NP_045009	hypothetical protein [Little cherry virus 1]	Closteroviridae; unclassified Closteroviridae
NP_047284	8.4 kDa protein [Rupestris stem pitting-associated virus]	Flexiviridae; Foveavirus
NP_049330	URF [Black beetle virus]	Nodaviridae; Alphanodavirus
NP_050005	ORF 0 [Sugarcane yellow leaf virus]	Luteoviridae; Polerovirus
NP_037580	ORF1 [Diaporthe ambigua RNA virus 1]	unclassified ssRNA positive-strand viruses
NP_037640	P6 protein [Barley yellow dwarf virus-PAS]	Luteoviridae; Luteovirus
NP_038456	movement protein (p8) [Japanese iris necrotic ring virus]	Tombusviridae; Carmovirus
NP_057949	lysis protein [Enterobacteria phage KU1]	Leviviridae; Levivirus; Enterobacteria phage BZ13
NP_058425	non-structural protein 3a [Transmissible gastroenteritis virus]	Nidovirales; Coronaviridae; Coronavirus; Group 1 species
NP_058429	non-structural protein 7 [Transmissible gastroenteritis virus]	Nidovirales; Coronaviridae; Coronavirus; Group 1 species
NP_059481	putative coat protein N-terminal extension [Chinese wheat mosaic virus]	Furovirus
NP_059939	unknown [cherry necrotic rusty mottle virus]	Flexiviridae; Foveavirus
NP_059941	7 kDa triple gene block protein [cherry necrotic rusty mottle virus]	Flexiviridae; Foveavirus
NP_059943	unknown [cherry necrotic rusty mottle virus]	Flexiviridae; Foveavirus
NP_062431	putative nucleic acid-binding protein [Cherry mottle leaf virus]	Flexiviridae; Trichovirus
NP_068345	p8 [Panicum mosaic virus]	Tombusviridae; Panicovirus
NP_619721	p7 protein [Maize chlorotic mottle virus]	Tombusviridae; Machlomovirus
NP_619720	p31 protein [Maize chlorotic mottle virus]	Tombusviridae; Machlomovirus
NP_619752	putative replicase [Oat chlorotic stunt virus]	Tombusviridae; Avenavirus
NP_705599	putative 2A; H-rev107 homolog; putative regulator of cell proliferation [Avian encephalomyelitis virus]	Picornaviridae; Hepatovirus; unclassified Hepatovirus
NP_705600	putative 2B; membrane-associated protein [Avian encephalomyelitis virus]	Picornaviridae; Hepatovirus; unclassified Hepatovirus
NP_705602	putative 3A; membrane-associated protein [Avian encephalomyelitis virus]	Picornaviridae; Hepatovirus; unclassified Hepatovirus
NP_705583	nsp1 (PCP1b); papain-like cysteine proteinase 1b preceeded by non-functional PCP1a domain [Equine arteritis virus]	Nidovirales; Arteriviridae; Arterivirus
NP_705589	nsp7 [Equine arteritis virus]	Nidovirales; Arteriviridae; Arterivirus
NP_705593	nsp12 [Equine arteritis virus]	Nidovirales; Arteriviridae; Arterivirus
NP_705594	nsp8 [Equine arteritis virus]	Nidovirales; Arteriviridae; Arterivirus
NP_705881	3A [Ljungan virus]	Picornaviridae; Parechovirus
NP_705882	3B; VPg [Ljungan virus]	Picornaviridae; Parechovirus
NP_714937	putative 3B [Simian enterovirus A]	Picornaviridae; Enterovirus
NP_733948	papain-like protease [Beet yellows virus]	Closteroviridae; Closterovirus
NP_734238	6K2 protein [Papaya ringspot virus]	Potyviridae; Potyvirus
NP_734332	6K2 protein [Tobacco vein mottling virus]	Potyviridae; Potyvirus

NP_734218	6K2 protein [Turnip mosaic virus]	Potyviridae; Potyvirus
NP_734073	Hypothetical protein [Bean pod mottle virus]	Comoviridae; Comovirus
NP_734005	VPg [Tomato ringspot virus]	Comoviridae; Nepovirus; Subgroup C
NP_734296	6k2 protein [Barley mild mosaic virus]	Potyviridae; Bymovirus
NP_734443	hypothetical protein [Parsnip yellow fleck virus]	Sequiviridae; Sequivirus
NP_734444	22.5 kDa coat protein [Parsnip yellow fleck virus]	Sequiviridae; Sequivirus
NP_734446	31 kDa coat protein [Parsnip yellow fleck virus]	Sequiviridae; Sequivirus
NP_734449	3C-like protease [Parsnip yellow fleck virus]	Sequiviridae; Sequivirus
NP_734403	6K2 protein [Johnsongrass mosaic virus]	Potyviridae; Potyvirus
NP_736601	VPg [Rice yellow mottle virus]	Sobemovirus
NP_734286	6K1 protein [Sweet potato mild mottle virus]	Potyviridae; Ipomovirus
NP_734289	6K2 protein [Sweet potato mild mottle virus]	Potyviridae; Ipomovirus
NP_733976	movement protein [Satsuma dwarf virus]	Sadwavirus
NP_733978	small capsid protein [Satsuma dwarf virus]	Sadwavirus
NP_734024	cysteine protease [Satsuma dwarf virus]	Sadwavirus
NP_734043	VPg [Blackcurrant reversion virus]	Comoviridae; Nepovirus; Subgroup C
NP_734020	VPg [Apple latent spherical virus]	Cheravirus
NP_736586	VPg [Ryegrass mottle virus]	Sobemovirus
NP_734440	P2 protein [Oat mosaic virus]	Potyviridae; Bymovirus
NP_733952	VPg [Strawberry mottle virus]	Sadwavirus
NP_733953	protease [Strawberry mottle virus]	Sadwavirus
NP_733983	movement protein [Strawberry mottle virus]	Sadwavirus
NP_733985	small capsid protein [Strawberry mottle virus]	Sadwavirus
NP_734394	6K2 protein [Cocksfoot streak virus]	Potyviridae; Potyvirus
NP_740662	capsid protein (C) [Rubella virus]	Togaviridae; Rubivirus
NP_740663	glycoprotein E2 [Rubella virus]	Togaviridae; Rubivirus
NP_740664	glycoprotein E1 [Rubella virus]	Togaviridae; Rubivirus
NP_740621	coronavirus nsp8 [Avian infectious bronchitis virus]	Nidovirales; Coronaviridae; Coronavirus
NP_740634	leader protein p87 [Avian infectious bronchitis virus]	Nidovirales; Coronaviridae; Coronavirus
NP_740453	VPg(3B) [Bovine enterovirus]	Picornaviridae; Enterovirus
NP_740553	2A mature peptide [Hepatitis A virus]	Picornaviridae; Hepatovirus
NP_740554	2B mature peptide [Hepatitis A virus]	Picornaviridae; Hepatovirus
NP_740556	3A mature peptide [Hepatitis A virus]	Picornaviridae; Hepatovirus
NP_740557	3B (VPg) mature peptide [Hepatitis A virus]	Picornaviridae; Hepatovirus
NP_740406	protein 2A [Encephalomyocarditis virus]	Picornaviridae; Cardiovirus
NP_740510	VPg1 protein [Foot-and-mouth disease virus C]	Picornaviridae; Aphthovirus; Foot-and-mouth disease virus
NP_740398	P-3A polypeptide [Human rhinovirus A]	Picornaviridae; Rhinovirus
NP_740594	nsp8 [Porcine reproductive and respiratory syndrome virus]	Nidovirales; Arteriviridae; Arterivirus
NP_740605	nsp12 [Porcine reproductive and respiratory syndrome virus]	Nidovirales; Arteriviridae; Arterivirus
NP_740380	3A [Equine rhinitis A virus]	Picornaviridae; Aphthovirus
NP_740381	3B (VPg) [Equine rhinitis A virus]	Picornaviridae; Aphthovirus
NP_740348	L protein [Porcine teschovirus]	Picornaviridae; Teschovirus
NP_740349	VP4 protein [Porcine teschovirus]	Picornaviridae; Teschovirus
NP_740354	2B protein [Porcine teschovirus]	Picornaviridae; Teschovirus
NP_740356	3A protein [Porcine teschovirus]	Picornaviridae; Teschovirus
NP_740357	3B protein [Porcine teschovirus]	Picornaviridae; Teschovirus
NP_740719	mature capsid protein gamma [Pariacoto virus]	Nodaviridae; Alphanodavirus
NP_740545	3B(VPg) [Human enterovirus B]	Picornaviridae; Enterovirus
NP_740591	VPg (3B) protein [Human enterovirus C]	Picornaviridae; Enterovirus
NP_740475	genome linked protein VPg [Poliovirus]	Picornaviridae; Enterovirus
NP_740523	3B (VPg) [Human rhinovirus B]	Picornaviridae; Rhinovirus
NP_740423	leader peptide [Theilovirus]	Picornaviridae; Cardiovirus
NP_740428	protein 2A [Theilovirus]	Picornaviridae; Cardiovirus
NP_740735	VPg protein 3B [Human parechovirus]	Picornaviridae; Parechovirus
NP_740745	VPg 3B [Human enterovirus D]	Picornaviridae; Enterovirus
NP_751900	VP29 precursor [Human astrovirus]	Astroviridae; Mamastrovirus
NP_751901	VP26 precursor [Human astrovirus]	Astroviridae; Mamastrovirus
NP_751902	NSMP-1, non-structural mature protein 1; N-terminal orf1ab cleavage product [Human astrovirus]	Astroviridae; Mamastrovirus
NP_751904	NSMP-4, non-structural mature protein 4; C-terminal part of the orf1a product [Human astrovirus]	Astroviridae; Mamastrovirus
NP_751919	core protein; p21c [Hepatitis C virus]	Flaviviridae; Hepacivirus
NP_751921	E2 protein; viral envelope protein [Hepatitis C virus]	Flaviviridae; Hepacivirus
NP_751922	p7 protein [Hepatitis C virus]	Flaviviridae; Hepacivirus
NP_751925	NS4A protein [Hepatitis C virus]	Flaviviridae; Hepacivirus

NP_757347	papain-like protease 1 [Citrus tristeza virus]	Closteroviridae; Closterovirus
NP_757348	papain-like protease 2 [Citrus tristeza virus]	Closteroviridae; Closterovirus
NP_757354	putative E2 protein [Hepatitis GB virus B]	Flaviviridae; unclassified Flaviviridae
NP_757355	putative protein p7 [Hepatitis GB virus B]	Flaviviridae; unclassified Flaviviridae
NP_757356	putative NS2 protein [Hepatitis GB virus B]	Flaviviridae; unclassified Flaviviridae
NP_757358	putative NS4A protein [Hepatitis GB virus B]	Flaviviridae; unclassified Flaviviridae
NP_758445	VPg genome-linked peptide [Sugarcane yellow leaf virus]	Luteoviridae; Polerovirus
NP_758538	3B (VPg) [Porcine enterovirus B]	Picornaviridae; Enterovirus
NP_776023	putative core protein C [Tamana bat virus]	Flaviviridae; Flavivirus; unclassified Flavivirus
NP_776024	putative anchored core protein C [Tamana bat virus]	Flaviviridae; Flavivirus; unclassified Flavivirus
NP_776025	putative preM protein [Tamana bat virus]	Flaviviridae; Flavivirus; unclassified Flavivirus
NP_776026	putative matrix protein M [Tamana bat virus]	Flaviviridae; Flavivirus; unclassified Flavivirus
NP_776028	putative non-structural protein NS1 [Tamana bat virus]	Flaviviridae; Flavivirus; unclassified Flavivirus
NP_776029	putative non-structural protein NS2a [Tamana bat virus]	Flaviviridae; Flavivirus; unclassified Flavivirus
NP_776030	putative non-structural protein NS2b [Tamana bat virus]	Flaviviridae; Flavivirus; unclassified Flavivirus
NP_776032	putative non-structural protein NS4a [Tamana bat virus]	Flaviviridae; Flavivirus; unclassified Flavivirus
NP_776033	putative 2K protein [Tamana bat virus]	Flaviviridae; Flavivirus; unclassified Flavivirus
NP_776034	putative non-structural protein NS4b [Tamana bat virus]	Flaviviridae; Flavivirus; unclassified Flavivirus
NP_776036	putative core protein C [Cell fusing agent virus]	Flaviviridae; Flavivirus; unclassified Flavivirus
NP_776037	putative anchored core protein C [Cell fusing agent virus]	Flaviviridae; Flavivirus; unclassified Flavivirus
NP_783303	p5.6 [Feline calicivirus]	Caliciviridae; Vesivirus
NP_786904	putative N-terminal leader protein [Canine calicivirus]	Caliciviridae; unclassified Caliciviridae
NP_803214	putative NS4A protein [Hepatitis GB virus A]	Flaviviridae; unclassified Flaviviridae
NP_803427	p150 [Rubella virus]	Togaviridae; Rubivirus
NP_828860	nsp1-pp1a/pp1ab [SARS coronavirus]	Nidovirales; Coronaviridae; Coronavirus
NP_828861	nsp2-pp1a/pp1ab [SARS coronavirus]	Nidovirales; Coronaviridae; Coronavirus
NP_937771	capsid protein [Kamiti River virus]	Flaviviridae; Flavivirus; unclassified Flavivirus
NP_937772	membrane protein [Kamiti River virus]	Flaviviridae; Flavivirus; unclassified Flavivirus
NP_937968	leader [Simian picornavirus 1]	Picornaviridae; unclassified Picornaviridae
NP_937981	small capsid protein [Euprosterna elaeasa virus]	Tetraviridae; Betatetravirus
NP_937982	small putative peptide [Euprosterna elaeasa virus]	Tetraviridae; Betatetravirus
NP_942000	VPg [Turnip rosette virus]	Sobemovirus
NP_942019	VPg [Cocksfoot mottle virus]	Sobemovirus
NP_945126	p12 protein [Pelargonium flower break virus]	Tombusviridae; Carmovirus
NP_951028	genome-linked protein [Broad bean wilt virus 1]	Comoviridae; Fabavirus
NP_982340	6 kDa protein 2 [Chilli veinal mottle virus]	Potyviridae; Potyvirus
YP_053926	hypothetical peptide [Tobacco ringspot virus]	Comoviridae; Nepovirus; Subgroup A
NP_734015	VPg [Cycas necrotic stunt virus]	Comoviridae; Nepovirus; Subgroup B
YP_054441	VPg [Arabis mosaic virus]	Comoviridae; Nepovirus; Subgroup A
NP_734036	VPg [Grapevine fanleaf virus]	Comoviridae; Nepovirus; Subgroup A
YP_081452	genome-linked viral protein [Cherry rasp leaf virus]	Cheravirus
NP_001006603	nonstructural protein 2K [Langat virus]	Flaviviridae; Flavivirus; tick-borne encephalitis virus group
YP_164820	leader [Duck picornavirus TW90A]	Picornaviridae; unclassified Picornaviridae
YP_227369	protease cofactor [Strawberry latent ringspot virus]	Sadwavirus
YP_227372	protease [Strawberry latent ringspot virus]	Sadwavirus
YP_227368	109 kDa polyprotein [Strawberry latent ringspot virus]	Sadwavirus
YP_227374	putative 40 kDa movement protein [Strawberry latent ringspot virus]	Sadwavirus
YP_227375	large coat protein [Strawberry latent ringspot virus]	Sadwavirus
YP_227376	small coat protein [Strawberry latent ringspot virus]	Sadwavirus
YP_238480	p6 [Pelargonium line pattern virus]	Tombusviridae; unclassified Tombusviridae
YP_308880	6K1 protein [Cucumber vein yellowing virus]	Potyviridae; unclassified Potyviridae
YP_308882	6K2 protein [Cucumber vein yellowing virus]	Potyviridae; unclassified Potyviridae
NP_056781	Y domain [Hepatitis E virus]	Hepeviridae; Hepevirus
NP_056782	papain-like protease [Hepatitis E virus]	Hepeviridae; Hepevirus
NP_056783	poly-proline hinge [Hepatitis E virus]	Hepeviridae; Hepevirus
NP_038457	movement protein (p12) [Japanese iris necrotic ring virus]	Tombusviridae; Carmovirus
NP_062884	structural polyprotein precursor [Rubella virus]	Togaviridae; Rubivirus

Supplementary Table S2: Taxonomic distribution of proteins for which at least one method finds homologs in more than one genus

For each sequence similarity search program, the taxonomic distribution of homologs is presented as follows: species/genus/family level. For instance, in the first row, in column Psi-Blast, 706/55/18 means that Psi-blast finds homologs of the query protein in 706 species, 55 genera, and 18 families.

The last column indicates the PFAM families or clans (groupings of homologous families) detected by HHpred as being homologous to the query, separated by semi-columns. If a PFAM family is part of a clan, that clan is indicated between brackets.

"?" means that there was no information available regarding the taxonomical distribution of hits. Reasons include the fact that no viral homolog was found (for HHpred), or that the taxonomic assignment is incomplete.

Accession	HHpred	HHblits	PSIBLAST	BLAST	Pfam (fam1 [clan1]; fam2 [clan2])
NP_573541	1579/151/47	4/1/1	706/55/18	4/1/1	Birna_RdRp; RdRP_1 [RdRP]
NP_803427	551/66/22	93/13/7	42/5/4	1/1/1	Peptidase_C27 [Peptidase_CA]; Macro [MACRO]; Rubi_NSP_C
NP_705599	516/64/22	34/6/4	1/1/1	1/1/1	LRAT [Peptidase_CA]; Calici_PP_N; NLPC_P60 [Peptidase_CA]; DUF830 [Peptidase_CA]; DUF778
NP_786945	516/64/22	23/5/3	5/3/1	3/2/1	Calici_PP_N; LRAT [Peptidase_CA]; NLPC_P60 [Peptidase_CA]; DUF830 [Peptidase_CA]; DUF778
NP_056788	654/72/21	61/3/2	154/11/3	11/2/2	SP2 [Viral_ssRNA_CP]; Astro_capsid; Viral_coat [Viral_ssRNA_CP]
NP_757347	511/61/21	8/1/1	8/1/1	2/1/1	DUF3648; DUF3762; Peptidase_C42 [Peptidase_CA]
NP_757348	511/61/21	8/1/1	8/1/1	1/1/1	DUF3614; Peptidase_C42 [Peptidase_CA]
NP_733948	511/61/21	8/1/1	6/1/1	2/1/1	Peptidase_C42 [Peptidase_CA]; DUF3614
NP_740719	608/71/20	7/1/1	1/1/1	1/1/1	Peptidase_A6 [Viral_ssRNA_CP]
NP_937981	608/71/20	2/1/1	2/1/1	2/1/1	Peptidase_A21 [Viral_ssRNA_CP]
NP_919038	608/71/20	2/?/1	2/?/1	2/?/1	Viral_coat [Viral_ssRNA_CP]
NP_786949	395/57/20	20/6/1	4/3/1	3/2/1	Peptidase_C37 [Peptidase_PA]; Peptidase_C24 [Peptidase_PA]; Peptidase_C3 [Peptidase_PA]; Trypsin_2 [Peptidase_PA]; Peptidase_S32 [Peptidase_PA]
NP_786948	395/57/20	20/6/1	3/2/1	3/2/1	Peptidase_C37 [Peptidase_PA]
NP_751904	395/57/20	22/2/1	10/1/1	10/1/1	DUF3621; Peptidase_C37 [Peptidase_PA]
NP_786947	395/57/20	3/2/1	3/2/1	3/2/1	Peptidase_C37 [Peptidase_PA]
NP_733953	395/57/20	2/?/1	2/?/1	2/?/1	Peptidase_C3 [Peptidase_PA]
YP_164806	341/43/15	29/9/7	1/1/1	1/1/1	OrfB_Zn_ribbon [Zn_Beta_Ribbon]; zf-NADH-PPase [Zn_Beta_Ribbon]; FYDLN_acid; UPF0547 [Zn_Beta_Ribbon]; DUF1610 [Zn_Beta_Ribbon]; TF_Zn_Ribbon [Zn_Beta_Ribbon]; PhnA_Zn_Ribbon [Zn_Beta_Ribbon]; zf-ribbon_3 [Zn_Beta_Ribbon]
NP_689397	66/22/13	60/18/8	43/11/6	1/1/1	Ribonuclease_3; dsrm [DSRM]
NP_056781	260/40/12	2/1/1	2/1/1	2/1/1	Vmethyltransf
NP_624334	94/16/6	1/1/1	1/1/1	1/1/1	MP
NP_734440	54/9/3	4/1/1	3/1/1	1/1/1	TMV_coat
NP_813800	62/8/3	39/3/1	42/3/1	7/1/1	Viral_Hsp90; Closter_coat; Tricho_coat
NP_891568	62/8/3	39/3/1	42/3/1	2/1/1	Viral_Hsp90; Closter_coat; Tricho_coat
YP_164263	57/6/3	57/6/3	56/6/3	2/1/1	Viral_NABP [Viral_NABP]; Carla_C4 [Viral_NABP]; CTV_P23 [Viral_NABP]
NP_203558	57/6/3	56/5/2	1/1/1	1/1/1	Viral_NABP [Viral_NABP]; Carla_C4 [Viral_NABP]
NP_062431	57/6/3	55/4/2	2/1/1	2/1/1	Viral_NABP [Viral_NABP]; Carla_C4 [Viral_NABP]
NP_619666	57/6/3	4/1/1	6/1/1	3/1/1	Viral_NABP [Viral_NABP]
YP_263306	87/5/2	80/5/2	51/3/2	1/1/1	7kD_coat
NP_059941	87/5/2	78/5/2	2/?/1	2/?/1	7kD_coat
NP_619562	87/5/2	77/5/2	47/3/2	1/1/1	7kD_coat

YP_277432	87/5/2	77/5/2	46/3/2	2/1/1	7kD_coat
NP_056756	87/5/2	76/5/2	43/3/2	1/1/1	7kD_coat
YP_054410	87/5/2	76/5/2	4/1/1	1/1/1	7kD_coat
NP_077082	87/5/2	76/5/2	3/1/1	3/1/1	7kD_coat
NP_112032	87/5/2	75/5/2	4/?/1	1/?/1	7kD_coat
NP_203556	87/5/2	74/5/2	1/1/1	1/1/1	7kD_coat
NP_620718	87/5/2	74/5/2	1/1/1	1/1/1	7kD_coat
YP_224137	87/5/2	71/5/2	1/1/1	1/1/1	7kD_coat
NP_624316	87/5/2	69/5/2	1/?/1	1/?/1	7kD_coat
NP_958174	87/5/2	75/4/2	49/4/2	8/1/1	7kD_coat
NP_663727	87/5/2	75/4/2	8/1/1	2/1/1	7kD_coat
YP_224087	87/5/2	75/4/2	1/1/1	1/1/1	7kD_coat
NP_604467	87/5/2	71/4/2	47/3/2	8/2/1	7kD_coat
NP_054028	87/5/2	71/4/2	8/1/1	1/1/1	7kD_coat
NP_620839	87/5/2	70/3/2	1/1/1	1/1/1	7kD_coat
NP_702991	87/5/2	70/3/2	1/1/1	1/1/1	7kD_coat
NP_044333	87/5/2	68/3/2	1/1/1	1/1/1	7kD_coat
YP_164261	87/5/2	60/3/2	1/1/1	1/1/1	7kD_coat
NP_040991	87/5/2	2/1/1	1/1/1	1/1/1	7kD_coat
NP_042586	87/5/2	2/1/1	1/1/1	1/1/1	7kD_coat
NP_619749	87/5/2	1/1/1	1/1/1	1/1/1	7kD_coat
NP_620645	87/5/2	1/1/1	1/1/1	1/1/1	7kD_coat
NP_047284	87/5/2	1/1/1	1/1/1	1/1/1	7kD_coat
NP_068550	19/5/2	1/1/1	1/1/1	1/1/1	Tricho_coat
NP_751901	48/3/2	46/2/2	68/2/1	14/1/1	Astro_capsid
NP_751900	48/3/2	11/2/2	34/1/1	5/1/1	Astro_capsid
NP_734238	97/7/1	81/2/1	87/2/1	3/1/1	Poty_PP
NP_734218	97/7/1	81/2/1	85/2/1	4/1/1	Poty_PP
NP_734332	97/7/1	81/2/1	85/2/1	1/1/1	Poty_PP
NP_734403	97/7/1	81/2/1	2/1/1	1/1/1	Poty_PP
NP_734394	97/7/1	81/2/1	1/1/1	1/1/1	Poty_PP
NP_982340	97/7/1	80/2/1	87/2/1	4/1/1	Poty_PP
NP_740349	12/6/1	12/6/1	1/1/1	1/1/1	VP4_2
NP_734286	81/5/1	1/1/1	5/1/1	1/1/1	Potyvirid-P3
NP_068344	22/4/1	4/3/1	2/2/1	2/2/1	MNSV_P7B; Tombus_movement
NP_068345	22/4/1	2/2/1	1/1/1	1/1/1	Tombus_movement
NP_620722	22/4/1	17/1/1	2/1/1	1/1/1	Tombus_movement
NP_613269	22/4/1	16/1/1	2/1/1	2/1/1	Tombus_movement
NP_619674	22/4/1	16/1/1	1/1/1	1/1/1	Tombus_movement
NP_038456	22/4/1	1/1/1	1/1/1	1/1/1	Tombus_movement
YP_077185	17/3/1	17/3/1	18/3/1	1/1/1	Luteo_PO
NP_114360	17/3/1	17/3/1	11/1/1	2/1/1	Luteo_PO
NP_604482	11/3/1	11/3/1	5/2/1	1/1/1	Viral_P18
NP_056822	7/3/1	6/3/1	5/2/1	5/2/1	RNA_capsid
NP_828854	33/2/1	33/2/1	3/1/1	3/1/1	NS3_envE
NP_041195	20/2/1	1/1/1	1/1/1	1/1/1	Ilar_coat
NP_740398	19/2/1	24/2/1	24/2/1	15/1/1	P3A
NP_740486	19/2/1	24/2/1	2/2/1	2/2/1	P3A
NP_054008	6/2/1	7/2/1	8/2/1	2/1/?	Umbravirus_LDM
YP_077279	2/2/1	2/2/1	2/2/1	2/2/1	DUF1478
NP_828861	6/1/1	24/2/1	25/2/1	7/1/1	DUF3477
NP_862838	3/1/1	20/3/1	2/1/1	1/1/1	MNSV_P7B
NP_044735	3/1/1	18/3/1	1/1/1	1/1/1	MNSV_P7B
NP_705881	2/1/1	3/2/1	2/1/1	1/1/1	Picorna_P3A
NP_740423	1/1/1	9/5/1	1/1/1	1/1/1	VP_N-CPKC
NP_705882	1/1/1	3/2/1	1/1/1	1/1/1	Parecho_VpG
NP_740735	1/1/1	3/2/1	1/1/1	1/1/1	Parecho_VpG
NP_740553	1/1/1	2/2/1	1/1/1	1/1/1	DUF3840
NP_740406	?/?/?	9/5/1	2/1/1	1/1/1	
NP_740428	?/?/?	9/5/1	2/1/1	1/1/1	
NP_740545	?/?/?	23/2/1	9/1/1	3/1/1	
NP_740591	?/?/?	23/2/1	5/1/1	1/1/1	
NP_740745	?/?/?	23/2/1	5/1/1	1/1/1	
NP_740475	?/?/?	23/2/1	3/1/1	1/1/1	
NP_758538	?/?/?	23/2/1	1/1/1	1/1/1	
NP_937968	?/?/?	21/2/1	1/1/1	1/1/1	

NP_068347	?/?/?	2/2/1	2/2/1	2/2/1
NP_705602	?/?/?	2/2/1	1/1/1	1/1/1
NP_740554	?/?/?	2/2/1	1/1/1	1/1/1
NP_740556	?/?/?	2/2/1	1/1/1	1/1/1
